# Future Directions for Chemosensory Connectomes: Best Practices and Specific Challenges

**DOI:** 10.3389/fnsys.2022.885304

**Published:** 2022-05-30

**Authors:** Maria G. Veldhuizen, Cinzia Cecchetto, Alexander W. Fjaeldstad, Michael C. Farruggia, Renée Hartig, Yuko Nakamura, Robert Pellegrino, Andy W. K. Yeung, Florian Ph. S. Fischmeister

**Affiliations:** ^1^Department of Anatomy, Faculty of Medicine, Mersin University, Mersin, Turkey; ^2^Department of General Psychology, University of Padova, Padua, Italy; ^3^Flavour Clinic, Department of Otorhinolaryngology, Regional Hospital West Jutland, Holstebro, Denmark; ^4^Interdepartmental Neuroscience Program, Yale University, New Haven, CT, United States; ^5^Department of Psychiatry and Psychotherapy, University Medical Center, Johannes Gutenberg University of Mainz, Mainz, Germany; ^6^Max Planck Institute for Biological Cybernetics, Tübingen, Germany; ^7^Functional and Comparative Neuroanatomy Laboratory, Werner Reichardt Centre for Integrative Neuroscience, Eberhard Karls University of Tübingen, Tübingen, Germany; ^8^The Graduate School of Arts and Sciences, The University of Tokyo, Tokyo, Japan; ^9^Monell Chemical Senses Center, Philadelphia, PA, United States; ^10^Oral and Maxillofacial Radiology, Applied Oral Sciences and Community Dental Care, Faculty of Dentistry, The University of Hong Kong, Pokfulam, Hong Kong SAR, China; ^11^Institute of Psychology, University of Graz, Graz, Austria; ^12^Department of Biomedical Imaging and Image-Guided Therapy, Medical University of Vienna, Vienna, Austria; ^13^BioTechMed-Graz, Graz, Austria

**Keywords:** chemosensory perception, functional magnetic resonance imaging – fMRI, good practice, connectome analysis, challenges and recommendations, study design and reporting

## Abstract

Ecological chemosensory stimuli almost always evoke responses in more than one sensory system. Moreover, any sensory processing takes place along a hierarchy of brain regions. So far, the field of chemosensory neuroimaging is dominated by studies that examine the role of brain regions in isolation. However, to completely understand neural processing of chemosensation, we must also examine interactions between regions. In general, the use of connectivity methods has increased in the neuroimaging field, providing important insights to physical sensory processing, such as vision, audition, and touch. A similar trend has been observed in chemosensory neuroimaging, however, these established techniques have largely not been rigorously applied to imaging studies on the chemical senses, leaving network insights overlooked. In this article, we first highlight some recent work in chemosensory connectomics and we summarize different connectomics techniques. Then, we outline specific challenges for chemosensory connectome neuroimaging studies. Finally, we review best practices from the general connectomics and neuroimaging fields. We recommend future studies to develop or use the following methods we perceive as key to improve chemosensory connectomics: (1) optimized study designs, (2) reporting guidelines, (3) consensus on brain parcellations, (4) consortium research, and (5) data sharing.

## Introduction

**FIGURE 1:**
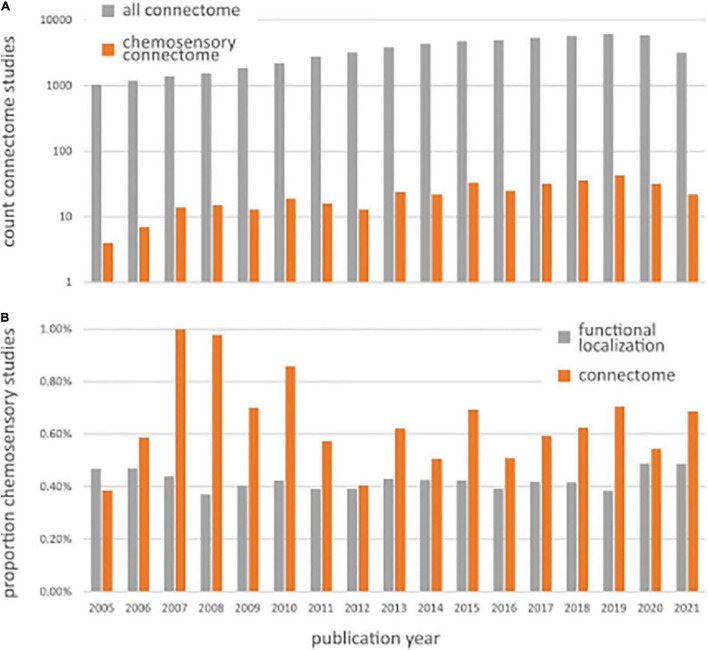
**(A)** Number of Pubmed search results for chemosensory connectomes (orange bars), returned from the query “human AND (chemosensory OR olfaction OR gustation) AND (connectome OR connectivity) AND (neuroimaging OR brain OR fMRI OR EEG OR MEG)” and all connectomes (gray bars), returned from the query “human AND (connectivity OR connectome) AND (neuroimaging OR fMRI OR brain OR EEG OR MEG).” **(B)** Chemosensory neuroimaging studies expressed as a proportion of the total number of studies using general neuroimaging methods (gray bars), returned from query “human AND (neuroimaging OR fMRI OR brain OR EEG OR MEG)” and chemosensory connectome studies expressed as a proportion of the total number of studies using connectome methods (gray bars), returned from the query “human AND (connectivity OR connectome) AND (neuroimaging OR fMRI OR brain OR EEG OR MEG).”

According to Sporns et al. (2005) and Sporns (2011), the human connectome is “a comprehensive structural description of the network of elements and connections forming the human brain.” Depending on the type of observations, brain networks can be structural or functional. Accordingly, the description of these brain networks can result in structural connectivity, which describes anatomical connections linking a set of neural elements, or functional connectivity, which describes the temporal dependence of neuronal activity patterns across multiple brain regions (Van Den Heuvel and Pol, 2010; Sporns, 2013). In the last 20 years, there has been a tremendous increase in knowledge about brain connectivity, in particular, regarding functional connectivity (Craddock et al., 2015). Since the term connectome was coined in 2005 (Sporns et al., 2005), there has been an increase in human connectome studies, with ∼6,000 publications per year over the last few years (see Figure 1A).

A deeper knowledge of brain network organization is fundamental to explain and predict dynamic neuronal interactions and to understanding the role of brain dynamics in shaping cognition and behavior (Sporns, 2011, 2013). This applies to all sensory processing which is placed along a hierarchy of brain regions (Mesulam, 1998) including processing of ecological chemosensory stimuli (as encountered in a natural, uncontrolled non-laboratory environment), in particular since they almost always evoke responses in more than one sensory system. The chemical senses include: olfaction (sensation resulting from volatile chemicals detected through olfactory sensory neurons in the nasal cavity described as spicy, moldy, fruity, floral, herbaceous), gustation, including sweet, sour, salty, bitter, umami, and possibly more taste sensations elicited by molecules binding to receptors in the oral cavity, and chemesthesis (thermal, nociceptive, and tactile sensations elicited by environmental chemicals acting on oral and nasal mucosal surface). For instance, a chair, if stationary, can usually be identified with only the visual system by determining its shape, color, and other features (Essen et al., 1992). To identify an odor smelled at a distance, we typically need other cues and chemicals with taste and chemesthesis properties that often only come into contact with their sensory systems through multimodal mediums, such as food. In order to solve this simple olfactory task, a multimodal process is required, which extends over several brain areas commonly seen as independent and concerned with processing stimuli of different sensory modalities (Gottfried and Dolan, 2003; Seubert et al., 2010; Cecchetto et al., 2020; Rekow et al., 2021).

So far, the field of chemosensory neuroimaging is dominated by studies that examine the role of brain regions in isolation, with on average 7% (±SD 2.5%) of studies since 2005 mentioning connectivity in either their keywords, title, or abstract (for the detailed search terms used to come to these numbers, see Figure 1 legend). Chemosensory connectome studies have increased in a similar manner as general connectome studies, characterized by a power relation comparable to general connectome studies (Figure 1A). When expressing chemosensory functional localization studies as a proportion of all functional localization studies and chemosensory connectome studies as a proportion of all connectome studies over time (Figure 1B), we observe that chemosensory connectome studies usually occupy a similar, but slightly larger proportion; around 0.5–1% of all chemosensory modality studies. This means that connectome studies investigating chemosensory modalities are conducted with a frequency comparable to functional localization studies, if not slightly higher. Given the specific physiology of chemosensory modalities, and the multisensory nature and experiential connectedness of ecological chemosensory stimuli, this higher number of connectome studies for chemosensory perception shows that functional connectomics is seen as an important and viable way to further advance the field.

Indeed, even though these three heterogeneous sensory modalities: smell, taste, and chemesthesis have distinct peripheral mechanisms, ecological stimuli rarely trigger them in isolation and these sensory signals are integrated centrally (e.g., Small and Prescott, 2005; Lundström et al., 2011; Frasnelli et al., 2012; Prescott, 2012; Spence, 2016). A classical example is flavor, the combination of gustatory stimulation in the oral cavity and olfactory stimulation via the retronasal route (Murphy et al., 1977; Rozin, 1982; Shepherd, 2006). Flavor evokes a unitary percept, despite originating from the contributions of three sensory modalities. Chemosensory perception is enriched by information from other sensory modalities, memory, and cognition (see e.g., Gottfried et al. (2004), Seo and Hummel (2011)), but it also plays a crucial role in emotion regulation (e.g., Kontaris et al., 2020), social interaction (e.g., Lübke and Pause, 2015), nutrition, and overall well-being (e.g., McGann, 2017; Boesveldt and Parma, 2021). Functional connectome studies further help us to understand the central processing of chemosensory signals. Farruggia et al. (2022) provide a more systematic overview of the progress made from connectivity research related to chemosensory function in humans.

Before proceeding to the methodology and specific challenges, we would like to highlight some recent work in chemosensory connectomics to provide a sample of the variety of neuroimaging connectome techniques employed in the field. For one, Zhou et al. (2019) used *k*-means clustering techniques and whole-brain functional connectivity patterns to parcellate the primary olfactory cortex into distinct clusters. These results showed that the human primary olfactory cortex can be divided into subregions that anatomically correspond to the anterior olfactory nucleus, olfactory tubercle, and frontal and temporal piriform cortices. They then used these clusters to create a map of the networks of the human olfactory system (Zhou et al., 2019). In another study, Arnold et al. (2020) used region-of-interest (ROI) and whole-brain analyses on resting-state functional magnetic resonance imaging (rs-fMRI) data, of nearly 900 participants from the Human Connectome Project (HCP), to map the connectivity matrix of the olfactory network. The authors showed that the olfactory network is comprised of three subnetworks (sensory, limbic, and frontal) and presents strong small-world properties, or features of efficient, global network communication. A small-world network is additionally characterized by the average number of steps between nodes, clustering, and segregation of the network into local clusters. Recently, a study by Esposito et al. (2022) compared post-COVID patients with persistent olfactory loss to controls (that never had COVID and had a normal sense of smell) using the aforementioned human olfactory network parcellation derived from graph-theoretic analyses (Arnold et al., 2020). Those with olfactory loss had an increased degree and strength of anterior piriform connectivity compared to those without COVID. Variation in local efficiency and clustering of anterior piriform connectivity within the olfactory loss/COVID group was negatively related to olfactory function. Overall, this suggests an increased functional segregation of the anterior piriform node from the rest of the olfactory network, perhaps reflective of a neuroprotective mechanism (Esposito et al., 2022). These two studies show how a network defined from publicly available big data can be validated in a smaller study that includes chemosensory function measures, and which can then provide insights into dysfunction, each study building on the previous.

Involvement of both the amygdala and thalamus in representing individual differences in taste sensitivity has been observed in functional localization studies of central gustatory processing (Spetter et al., 2010; Yeung et al., 2016). Two decades earlier, Small et al. (2001) had already proposed the involvement of the amygdala in taste intensity perception, after observing that resection of the amygdala and surrounding tissue in epilepsy patients caused an increase in taste intensity ratings. This counterintuitive observation led to the prediction that the likely inhibitory influence of the amygdala on other gustatory cortical areas is responsible for central intensity regulation. A recent gustatory connectome study sought to identify this specific network involving the amygdala. Veldhuizen et al. (2020) first used responses to various taste qualities (sweet, sour, and salty) versus tasteless to identify the gustatory cortex. Taste responsive clusters were then used as seeds in psychophysiological interaction analyses (PPI, a technique that finds functionally connected areas across the entire brain) to pre-select gustatory cortical regions that correlate with individual differences in taste sensitivity (regardless of taste quality). Then, from those regions, a fully connected dynamic causal model (DCM, a technique that allows the construction of a complex connectivity parameters between a pre-determined set of regions) was specified, which was subsequently pruned to a sparsely connected model that best explained the observed data. In that optimal model, gustatory sensitivity correlates primarily with inhibitory connections from amygdala to thalamic areas. This is an example of neuroscientific evidence from various methodologies (neuropsychology, functional localization neuroimaging, and connectome analysis) converging onto a model that proposes an explanation for an important feature of gustatory perception.

Nonetheless, for advances to occur in emerging research topics from more specialized fields, there is a critical need for bidirectional evaluation of best practices and challenges between human chemosensory and connectome neuroimaging fields. Indeed, best practices from the general neuroimaging field are to be evaluated for their relevance and appropriateness in the chemosensory field. Further, specific challenges of chemosensory neuroimaging should be reviewed in the context of connectivity studies to identify any potential new challenges. Therefore, the aim of this article is twofold: (1) to communicate current existing neuroimaging and connectome guidelines to the chemosensory community and (2) to outline specific challenges and potential solutions for chemosensory studies. The scope here generally focuses on connectivity from mesoscopic/macroscopic imaging in humans, except when there is pertinent relevance from animal work.

## Methods to Study the Functional Connectome

The human brain and the relation between its regions can be understood using various distinct measures each describing a different, either structural or functional, aspect of the human connectome. Structural connectivity obtained through either high resolution anatomical or diffusion-weighted images allows the description of the mere static anatomical organization of the brain. Functional connectivity, on the other hand, aims to describe the temporal relationship between neurophysiological events observed at spatially separated brain regions (Friston, 1994). Commonly, functional connectivity is obtained by calculating the statistical similarity between two or more signals obtained using functional imaging (e.g., positron emission tomography, PET, or functional MRI) or electrophysiological methods like magneto- (MEG) or electro-encephalography (EEG). Methods to obtain this functional similarity can be roughly divided into model-dependent approaches, mainly represented by seed-based methods (Biswal et al., 1995; Lowe et al., 1998), and model-free or data-driven techniques, comprising all forms of clustering (Cordes et al., 2002; Van Den Heuvel et al., 2008) and decomposition approaches like PCA (Friston et al., 1993) and ICA (see e.g., Calhoun et al. (2001), Beckmann et al. (2005)). Finally, there are effective connectivity methods like structural equation modeling (e.g., Gates et al., 2011) and their variants as well as dynamic causal modeling (Friston et al., 2003; Friston, 2009; Friston, 2011). Effective connectivity methods try to describe the effect of one node over another given a certain network model and input or task (Friston, 2011). A complete overview of all these available methods is beyond the scope of this paper and thus we will restrict ourselves largely to model-based approaches. This limitation seems justified since seed-based approaches are the most common ways to describe the chemosensory neuronal network (Farruggia et al., 2022).

### Seed-Based Approaches

Seed-based approaches represent the most straightforward way to observe functional connectivity (Biswal et al., 1995; Lowe et al., 1998). Here, functional connectivity is defined by some measure of similarity, i.e., Pearson’s correlation, between the time series of two particular nodes in the brain resulting in some kind of functional connectivity map (Lowe et al., 2016). Typically, highly similar or correlated time series are interpreted as some kind of functional cooperation, or connectivity, between brain areas in relation to overt or covert behavior. However, it should be noted that these functional connectivity patterns may be influenced by simple changes in signal amplitude at one or more sources or by a third unknown input that drives the connection as a kind of confound (Duff et al., 2018). Additionally, this pattern may depend on the chosen measure since Pearson’s correlation only measures linear time-domain dependencies although higher-order dependencies or interdependencies may apply (Mohanty et al., 2020).

Furthermore, functional connectivity does not necessarily imply structural connectivity as neuronal information relay is multi-dimensional and integrative across several interacting structures (Friston, 2011). However, modeling results indicate that resting-state functional connectivity, particularly its strength, persistence, and spatial statistics, is constrained by the anatomical structure of the human cerebral cortex (see e.g., Honey et al. (2009)).

Although intriguingly simple, seed-based approaches pose some serious drawbacks that have to be addressed. Data for seed-based functional connectivity analyses may be collected during rs-fMRI where participants are given no particular task besides fixating on a visual cue, such as a cross on a screen. Up to now, there is no consensus on an optimum resting-state scan duration, but typically durations of around 6–10 min have been shown to produce reliable results (Van Dijk et al., 2010; Birn et al., 2014). Functional connectivity is, however, not restricted to resting-state data and can also be applied to task evoked imaging data. Yet, when calculating task-state functional connectivity, i.e., the statistical connection between the neural time-series when processing tasks, one has to take care for task-induced effects since they can systematically affect task-state functional connectivity (see Cole et al. (2019), for a comprehensive evaluation). Irrespective of the origin of the data, rest or task, non-neuronal sources have to be removed from the data before calculating functional connectivity. In particular, head movement and physiological signal confounds originating from respiration and cardiac activity may introduce major challenges (Power et al., 2012; Van Dijk et al., 2012). Data denoising steps and their possible impact on connectivity measures are well covered in the literature; see for example Murphy et al. (2013), Burgess et al. (2016), Satterthwaite et al. (2019), Power et al. (2020), and Kassinopoulos and Mitsis (2022) for possible reviews and evaluations on fMRI denoising. In later sections, we discuss denoising steps in relation to chemosensory perception in particular.

Definition and selection of seed (or node) regions for connectivity analyses represent their own problem and have developed into a completely separate line of research. Depending on the question at hand, seeds can be defined *a priori* using atlases or created through a functional activation map based on a separate task (Biswal et al., 1995; Lowe et al., 1998). However, such task-defined ROIs have to be used with caution and should not be employed to describe functional connectivity on a whole-brain scale (Van Den Heuvel and Pol, 2010). Over the past decade, there has been an increasing number of connectivity studies calculating correlations between time series of all units in a parcellation of the brain. These parcellations commonly try to summarize anatomical divisions based on some structural or functional commonality to generate a general atlas. One commonly known example is the automated anatomical parcellation (AAL; Tzourio-Mazoyer et al., 2002). Next to general connectome atlases, like the Schaefer atlas (Schaefer et al., 2018), integrating local gradient and global similarity approaches to defining neurobiologically plausible nodes, several specific and functional parcellation schemes exist for the human primary olfactory cortex (Zhou et al., 2019) or for the olfactory connectome as a whole (Arnold et al., 2020). We discuss the selection of nodes, or regions-of-interest, with respect to chemosensory perception within the next chapter in more detail.

Next to Pearson’s correlation, probably being the most popular and widely used measure, various other measures exist to describe the similarity between two or more nodes. Alternative measures mostly comprise extensions of Pearson’s correlation, like cross-correlation, representing the linear correlation between all possible shifted versions of two signals or coherence allowing to describe similarity in the frequency domain (c.f. Bastos and Schoffelen, 2016; Mohanty et al., 2020). While the latter is more commonly used to describe an oscillatory neuronal activity, multivariate and weighted connectivity, they employ semi partial correlation measures to control for mediator effects exerted from either other regions or a presented task. (Generalized) psychophysiological interaction analyses (Friston et al., 1997; Gitelman et al., 2003; McLaren et al., 2012) finally represent the task-modulated connectivity between two or more nodes in the brain, i.e., gPPI allows the examination of connectivity between the seed region and other spatial units under different psychological states.

Task-based connectivity can employ generalized linear models (GLMs) to correlate event-related BOLD signals, or modeled beta weights, between voxel- or region-wise beta series data (Rissman et al., 2004; Göttlich et al., 2015). Hartig et al. (in review) used an informed structural parcellation scheme for the gustatory connectome to study how basic taste stimuli interact amongst taste network regions. This work identified a strongly inter-connected, tri-modular network in healthy rhesus macaque monkeys, positing a translational primate taste network map.

All these measures can be used to describe connectivity on various levels throughout the entire brain. When interested in only a few separate regions and their connectivity with the rest of the brain, a simple seed-to-voxel analysis can be conducted. This technique is best used when interested in questions about function across the entire brain or when there are no *a priori* specified networks known (see e.g., Zhou et al. (2019)). Alternatively, seed-to-voxel can be used to study the behavior of known networks under differential conditions (see e.g., Cecchetto et al., 2019). Research questions that involve multiple *a priori* defined neuroanatomical nodes can be answered using an ROI-to-ROI connectivity analysis. This technique is similar to the seed-to-voxel approach, but characterizes the connectivity between all pairs of ROIs circumventing the asymmetry between seeds and voxel. ROI-to-ROI connectivity allows the specification of the circuitry within a probably known anatomical functional unit, including directional intrinsic connections between regions using multivariate regression coefficients, driving external inputs to the network (events or stimuli) and modulators of regions or connections (task or between-participant variables). Such an ROI-to-ROI analysis builds the basis for the olfactory connectome described by Arnold et al. (2020) and the gustatory connectome described by Hartig et al. (in review). Finally, one can describe the entire voxel-to-voxel connectome by calculating the connectivity between all pairs of, for example, voxels or anatomical units in the brain.

### Model-Free Approaches

Model-free approaches aim to overcome some of the limitations of seed-based methods and allow for the examination of whole-brain connectivity without the need for an *a priori* definition of regions-of-interest. As such, model-free approaches focus on the description of possibly unique connectivity patterns across the brain during resting-state as well as during task conditions. Independent component analysis (ICA) and its further developments is perhaps the main representative of these approaches (Calhoun and Adali, 2012). In short, ICA-based methods aim to present the underlying sources that build the current data by looking for maximally independent patterns. The development over time as well as the spatial pattern itself can then be readily used for further examination. The downside of this simple handling is that the derived connectivity patterns are often hard to interpret due to their inherently higher complexity as compared to seed-based functional connectivity, e.g., not all patterns identified are biologically plausible or even represent neuronal events. This may hamper the selection and interpretation of independent patterns as well as their translation into clinical relevance (Fox and Raichle, 2007). Nevertheless, ICA has shown to be comparable to seed-based functional connectivity, and is a reliable and replicable method to detect independent connectivity patterns (Damoiseaux et al., 2006; Smith et al., 2012; Noble et al., 2019); ICA is influential due to its availability for use in big open datasets, such as the UK Biobank (Sudlow et al., 2015) and Human Connectome Project (Van Essen et al., 2013).

### Investigating the Dynamics of the Network

Recently, several studies have started to examine the dynamic fluctuations of functional connectivity patterns across time, showing that a stationary approach may be too simplistic (e.g., Beckmann et al., 2005; Chang and Glover, 2010). Sliding window correlation techniques (e.g., Hindriks et al., 2016) can be employed to explore this varying nature and interactions within networks. Other available approaches are for example temporal independent component analysis (Smith et al., 2012), model-based approaches (c.f. Lindquist et al., 2014), or time-frequency coherence analysis (Chang and Glover, 2010).

Sliding window correlation analyses, representing the most popular strategy, describe fluctuations over time by segmenting the time course of neuronal nodes into sets of separate windows. Inside these time windows, all aforementioned described methods characterizing similarity can be applied. Despite its obvious simplicity, sliding window analyses require a large number of decisions affecting results and possibly rendering them uninterpretable. These include, among others, choice of preprocessing (see previous section on seed-based approaches), window length and shape (e.g., Leonardi and Van De Ville, 2015; Zalesky and Breakspear, 2015), and the selection, extraction, and interpretation of measures of similarity within a given time-window (see Preti et al. (2017), for a review). Similar reviews exist on dynamic functional connectivity in multimodal imaging (Tagliazucchi and Laufs, 2015), independent component analysis (Calhoun et al., 2014), and more generally on open issues and controversies (Lurie et al., 2020).

Within the field of chemosensory perception, dynamic connectivity methods are not well established probably due to the complexity of the field. Furthermore, it is unclear if the time window to look for shifts in connectivity may be appropriate for chemosensory studies at all. To overcome possible dependencies and biases originating from the choice of window length, authors commonly average across a set of predefined window lengths (Iravani et al., 2021a). However, detailed studies employing dynamic functional connectivity to assess the transition of chemosensory networks and their association with cognition, behavior, and clinical variables are still missing.

### Investigating the Organization of a Network

Large-scale connectivity matrices, regardless of how they are generated, closely resemble complex networks, with features like small-world properties as well as highly connected, modular hubs (Bullmore and Sporns, 2009). Graph theory is a versatile mathematical application to study the relationships between vertices or nodes, and the connection between them, their edges (Prathik et al., 2016). Such graphs are generated by thresholding large-scale functional connectivity maps. From this resulting graph, various network measures are derived, including strength of correlations, density of connections, centrality, and path length to summarize the properties of a network (see Rubinov and Sporns (2010)) for available measures and their interpretation.

Throughout recent years, graph theoretical approaches have proven an effective tool to characterize complex brain networks and aided the study of neural networks and connectomes in health (Sun et al., 2017; Hartig et al., in review) and disease (e.g., Parkinson’s disease see Fang et al. (2017), Kazeminejad et al. (2017)).

However, there are some caveats here to consider (Luo et al., 2021). First, one has to note the influence of data preprocessing on graph theory derived measures (see e.g., Gargouri et al., 2018 for a comprehensive evaluation). Of particular interest is the question of which graph represents the brain best. The answer to this question rests on three pillars, the definition of nodes, the definition of edges, and finally the matrix to describe the graph. The definition of nodes and edges resembles issues already mentioned before. Within graph-theoretic approaches, these decisions have a greater impact since they directly modify the database for all further analyses (Fornito et al., 2015).

Generally, edges between nodes of a graph can be undirected or directed, the latter of which indicates directionality between any two connected nodes. Typically, with fMRI an undirected graph is computed based on the correlation strength between time-series activity or modeled changes in event-related activity over time between pairs of nodes, or regions. Some caution may be warranted with using BOLD signal to develop directed graphs or derive causal inferences given the intrinsic signal latency in the indirect measurement of neural activity by hemodynamic coupled metabolic changes (Huettel et al., 2004).

Matrix thresholding, or the definition of edges building the graph, is another major topic since spurious and weak connections should be eliminated, while strong, robust connections of interest should be preserved (Garrison et al., 2015). Several approaches depending on the research interest at hand have been developed, ranging from consensus filtering to various multi-scale approaches (see Fornito et al. (2013), Zalesky et al. (2016), Van Den Heuvel et al. (2017)).

Finally, graph-theoretic analyses pose special statistical problems due to the binary nature of a graph. For the statistical evaluation of network-related parameters and measures derived from a graph a plethora of different models, ranging from simple t-tests to non-parametric tests to general linear model approaches, exist. However, surprisingly few methods exist to quantitatively compare two graphs originating for example from two different groups, conditions themselves (see Mheich et al. (2020), for possible approaches).

### Inferring Behavior From the Connectome

Previously described methods to measure connectivity, in a very strict sense, do not permit describing causal relations between brain activity and behavior. This is largely due to employed correlation or regression approach which have a tendency to overfit or incompletely represent the data (e.g., Mohanty et al., 2020). This can further hinder accurate predictions of behavior. Cross-validation, or the splitting of data into a training and a test set, represents a conservative way to establish brain-behavior relations reliably. Several methods exist that follow this approach, among which for example are connectome-based predictive modeling (CPM) (see below), support vector approaches (see Iravani et al. (2021a) for an application in olfaction) and other machine learning methods (Chacko et al., 2020; Lötsch et al., 2020).

Connectome-based predictive modeling is a tool used to predict behavior from whole-brain functional connectivity (Finn et al., 2015; Shen et al., 2017). At its most basic level, CPM involves several steps. Connectivity matrices are first created from the data, which are split into training and test sets. Then, matrix edges are regressed against behavioral measures, with the most significant edges retained for analysis. Significant edges are then added together to create a summary statistic, and these are finally fit in a linear model to predict behavior on the test set (Shen et al., 2017). CPM has been used to predict behavior in a variety of contexts, from anthropometric features, such as waist circumference (Farruggia et al., 2020), to personality traits (Hsu et al., 2018) and irritability in youth (Scheinost et al., 2021). Recently, CPM has also been used in chemosensory neuroimaging to discriminate between individuals with anosmia and normosmia (Pellegrino et al., 2021a). This technique, in combination with functional localization analysis, helped demonstrate that olfactory dysfunction resulting from traumatic injury coincides with differential activity outside of the olfactory cortex; namely, in prefrontal cortex, frontal operculum, and posterior cingulate cortex (Pellegrino et al., 2021a). This single study shows that CPM holds promise to aid our understanding of central chemosensory processes. Further analyses using new and novel datasets are warranted using this technique.

## Challenges to Chemosensory Connectome Neuroimaging Studies

Since the basis of connectome studies is, in essence, a measure of covariation of two variables over time, any factor that may drive covariation besides connectivity can be a confounding variable. In general, two types of influences are of major concern for connectivity studies in awake humans: non-neural physiological signals (i.e., respiration and cardiac activity) and movement. Additional challenges are inherent to chemosensory studies, such as the location of the regions-of-interest, which can suffer from distortion in imaging (Ojemann et al., 1997), the unknown appropriateness of conventional choices for data cleaning and model fitting parameters, neurophysiological aspects of the chemical senses, multisensory stimuli, task design, and sample size. We discuss these, and related issues, in turn and highlight where some of these variables intersect and pose a particular challenge for chemosensory connectome studies. We also suggest solutions and indicate where methodological studies are needed. This information is summarized in Table 1.

**TABLE 1:** Description of challenges to chemosensory connectome neuroimaging studies.

Domain	Variables	Solution	Intersects with
Physiological	• Respiration. • Cardiac.	• Measure and regress at single subject-level and group level.	Brain region Task Movement
Movement	• Inherent movement in ecological chemosensory stimulation.	• Minimize with training, physical restraint or tactile feedback. • Measure and regress at single subject-level and group level models.	Physiological Task
Data cleaning/model fitting	• Choice of high-pass filter. • Detrending/global signal regression. • Hemodynamic model appropriateness (GLM, DCM, and PPI).	• Adjust based on task design. • Perform with and without detrending and report. • Methodological studies needed.	Task Neurophys of chemical senses
Brain region	• Sparse representation. • Proximity to high distortion susceptibility areas. • Parcellation appropriateness.	• None, studies needed. • Optimized data collection. • Optimized atlases.	Task Physiological
Neurophysiology of chemical senses	• Adaptation and habituation. • Individual differences tend to be large in chemosensory studies.	• Optimize task design/interleaving stimuli. • Titrate stimuli and measure and regress at group level. • Standardize stimuli across studies.	Data cleaning/model fitting
Stimuli	• Most chemosensory stimuli are multisensory. • Long presentation times.	• Optimize stimulus choice. • Measure perception. • Control stimuli/conditions/experiments. • Block design.	Brain region
Task	• Resting-state usually less sensitive than task.	• Optimize task design for research question.	Brain region. Neurophys of chemical senses
Acoustic noise and visual input	• Scanner noise may change perception and mask other connectivity. • Eyes-open vs. eyes-closed different brain states.	• Mock scanning to habituate participants to the environment. • Provide uniform instructions for participants/report in method/verify compliance and use regressors.	
Sample size and brain-behavior relation	• Chemosensory studies tend to have low sample size	• Employ resting-state. • (re)use big datasets. • Collapse (public) data from multiple studies. • Include chemosensory tasks in future big studies.	Task Neurophys of chemical senses

### Physiological Noise

There are two important sources of physiological noise that may lead to biased estimates of connectivity: respiration and cardiac output. Arteries and sinuses are in proximity to many brain areas and may influence estimates of neural response. Specifically, medial frontal and temporal brain regions may be disproportionately affected due to their proximity to the sinus sagittalis and large arteries (Ojemann et al., 1997). Many chemosensory regions-of-interests are in the medial frontal and temporal lobes, therefore physiological noise may be of particular concern for chemosensory connectome studies.

**FIGURE 2:**
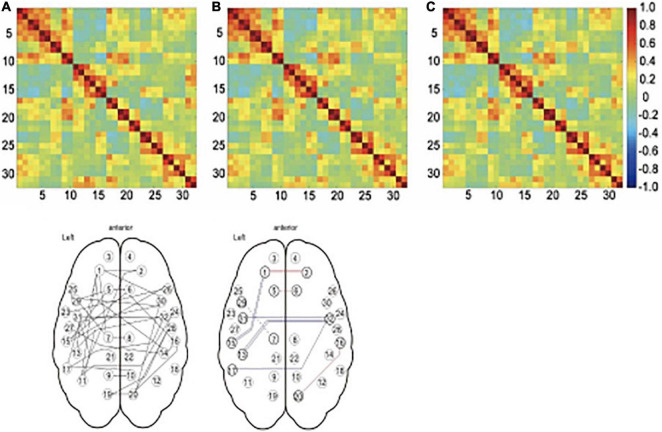
Effect of physiological noise regressors on connectivity. The top panel shows the connectivity matrices for 36 networks, including the default mode network and related brain regions. The color of each cell reflects the correlation coefficient, with red representing strong positive correlations, green representing no correlation, and blue representing strong negative correlations. The time series used to produce the matrix in **(A)** were not corrected for physiological noise, in **(B)**, the time series were corrected for cardiac noise, and in **(C)**, the time series were corrected for cardiac noise and respiratory noise. While these matrices do not look very different at first glance, there were significant differences in some of the connections as shown in D. Panel E illustrates which models differed in *post hoc t-test* between the models. The color of the line indicates the sign of the change, with red lines showing increased connectivity strength and blue lines showing decreased connectivity strength. A double line shows a significant difference between the uncorrected model and both of the models with corrected time series (panel **A** vs **B** and **C**), while a single unbroken line indicates the difference between the uncorrected model and model corrected for cardiac noise (panel **A** vs **B**) and a single broken line indicates the difference between the uncorrected model and the model corrected for both types of noise (panel **A** vs **C**). Reproduced from Yoshikawa et al. (2020).

Yoshikawa et al. (2020) examined the influence of respiration and pulse on functional connectivity by measuring respiratory flow with a nasal mask and pulse rate with an infrared sensor. Connectivity of the default-mode network (and related areas) was compared between three models including: (1) no physiological noise regressors, (2) a cardiac output regressor, and (3) both cardiac output and respiration regressors. In general, the default mode network (DMN) and related areas looked similar between the models, but the connectivity estimates between regions differed (see Figure 2). Specifically, medial frontal regions showed an increase in their connectivity strength when statistically accounting for cardiac rhythm, while temporal regions showed a decrease in their connectivity strength when correcting for respiration.

Vasodilation and vasoconstriction also leads to local and global changes in BOLD response, which can be a confounding factor (Chang et al., 2008). Ideally, cerebrovascular reactivity is measured and included as a regressor in the design and there are various suggestions on how to complement resting-state connectivity protocols with an estimate (Pinto et al., 2021; Stickland et al., 2021). These results show that chemosensory connectome studies may be biased in two ways if physiological noise is not accounted for; it may lead to both over- and under-estimates of connectivity.

Methodological studies that assess the influence of physiological confounding factors typically examine BOLD response measured with fMRI during resting-state only. Different concerns may arise for other mesoscopic/macroscopic neuroimaging types like Single-Photon Emission Computerized Tomography (SPECT), Near Infrared Spectroscopy (NIRS), Positron Emission Tomography (PET), Electroencephalography (EEG), and Magnetoencephalography (MEG). It is also important to note that physiological noise may be of greater concern in some task-related designs. For example, when using salient or emotional stimuli, arousal may induce respiratory and cardiac changes, which will then induce task-correlated physiological noise. Chemosensory stimuli are often experienced as salient and the primary dimension of perception is often pleasantness (Khan et al., 2007; Engen, 2012), and stimulus delivery may depend on in- or expiration. In addition, as the study by Yoshikawa et al. (2020) showed, head movement can be induced by respiration, which will exacerbate the confounding effects of physiological factors. Conversely, it must be noted that, while the chemosensory modalities might be more vulnerable to the confounding effects of non-neural activity, there is also the risk of losing relevant information. Neural signals can also encode physiological information and the chemosensory connectome may, in part, reflect the body’s physiological state. Therefore, the intersection of respiration, movement, task, and stimulus valence may be a particular biasing factor in chemosensory connectome studies.

### Movement

**FIGURE 3:**
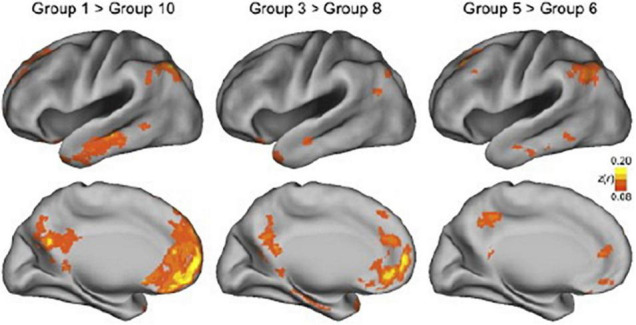
Effect of movement on connectivity maps. In this study, functional networks were estimated for groups of ∼100 participants each. The groups were created by ranking participants by head motion, with group 1 containing the 100 participants with the least movement and group 10 containing the 100 participants having the most movement. The top panels show the lateral view of the cerebral cortex, while the bottom panels show the medial view. The superimposed blobs indicate significant connectivity differences. The color indicates the z-value of the difference in connectivity estimates, with yellow indicating a greater difference and orange/red a smaller difference. The two maps on the left show the difference between the bottom 10 percentile (participants that moved least) and the top 10 percentile (participants that moved most). The middle and right panels also show differences between deciles, but progressively closer to each other in terms of movement. As can be seen, for large areas in the temporal, medial parietal, and medial frontal lobes, connectivity estimates are affected by movement. This figure also demonstrates that all comparisons show differences in connectivity estimates, even in the two most adjacent groups in the panel on the right. Reproduced from Van Dijk et al. (2012).

The most commonly recognized and perhaps strongest confounding factor of connectivity estimates in awake humans is movement. Participants move their heads involuntarily and voluntarily for many reasons, and movement occurs naturally when breathing (c.f. Yoshikawa et al., 2020). Generally, it is recommended to minimize movement by physically making movement less likely, for example, by the use of foam pillows that fill the open space between the head and transmit/receiver coils in fMRI studies or the use of a chin rest as used in EEG studies. An fMRI study directly examined the effect of movement by Van Dijk et al. (2012) and found that connectivity estimates (expectedly) differed between the top 10 and bottom 10 percentiles, but also between adjacent groups that showed very small, but systematic differences in movement (Figure 3). The groups with more movement showed reduced functional connectivity in whole-brain distributed networks (i.e., the default mode network) and increased connectivity in local networks. Note that in this study participants were restrained with a foam pillow and extendable padded head clamps, so the ability to move was already minimized. Similar results were found by Power et al. (2012) and resulted in the description of framewise displacement, a measure to index between volume movement (Power et al., 2011). Already these findings show the induced variation if movement is not reduced and accounted for; it may lead again to both over- and under-estimates of connectivity.

Head movement estimates are both heritable and stable over time within participants, indicating that some people just move more, raising concern for studies of individual differences (Hodgson et al., 2017). Head movement is also a major concern in studies that compare groups of participants since it is known that between-participant factors are related to head movement. For example, age, BMI, neurological disease, and psychiatric diagnosis are all positively correlated with head movement (Zeng et al., 2014; Hodgson et al., 2017). Head movement may pose a particular challenge for chemosensory connectome studies using a task design (as opposed to resting-state), since stimulus delivery may depend on in- or expiration or swallowing when doing taste studies.

Thus, it is important for chemosensory connectome studies to account for the head movement of each individual (to account for within-participant variance of each regressor) as well as at the group level (to account for individual differences between participants). It is also recommended that researchers take additional measures beyond the usual to constrain movement (within the boundaries of comfort naturally). Some gustatory studies have used a bite bar (Haase et al., 2007) and others have used a simple sticky tape or soft velcro band across the forehead (Krause et al., 2019; Jolly et al., 2020). This last method gives the participant tactile feedback on movement without actively restraining them. There is some evidence that custom-molded head cases make movement during talking worse, and we anticipate a similar effect with swallowing (Jolly et al., 2020). Training with feedback on movement in an operant conditioning task can also be helpful (Voos and Pelphrey, 2013).

Methodological studies investigating the exact effect of choices to reduce movement are necessary. For example, for gustatory studies it is unknown how excluding (by suctioning out liquid) or postponing swallowing (beyond the HRF window time-locked to stimulus delivery) affects the results. Similarly, it is unclear what the effect is of gating olfactory stimulus delivery by inhalation through the nose or what effect isolating respiration from olfactory stimulus delivery by velopharyngeal closure has on neural responses (Sobel et al., 1998). This type of strict control over movement may be important for a complete understanding of isolated processes. However, it would certainly be a very artificial state of sensory stimulation, as isolating gustatory stimulation from swallowing and olfactory stimulation from sniffing would not occur naturally very often. To avoid even more movement than from swallowing alone, neuroimaging of flavor has so far been entirely restricted to liquids only, so chewing is not part of the process of stimulation. This is an important limitation to ecological validity, as most of the foods we consume are chewed before swallowing. As has also been suggested by Smeets et al. (2019), perhaps future use of advanced imaging protocols, with for example multi-echo imaging (Kundu et al., 2017) and head-movement tracking with sub-millimeter resolution (Weisenberger et al., 2006) will make this possible.

### Brain Region

Most regions-of-interest for chemosensory connectome studies are in (para)limbic and subcortical areas, not only in the neocortex. These regions have a different neurophysiology and, due to their location in the brain (close to sinuses and arteries), they are typically more susceptible to various distortion factors than cerebral cortical areas (Ojemann et al., 1997). Limbic and paralimbic cortices are considered intermediaries between subcortical and cerebral cortical areas, and neural responses here are characterized by a lack of segregation by modality of sensory input (Mesulam, 1998). For example, the dorsal granular insular cortex, a direct recipient of afferent projections from the taste thalamus (Pritchard et al., 1986), is characterized by sparseness and multimodality of taste-responsiveness, with only around 5% of neurons responding to taste stimuli (Smith-Swintosky et al., 1991). Of those neurons, only about half show unimodal taste responses, while the rest also respond to other oral sensations, like temperature and/or texture (Verhagen et al., 2004) or mouth movement (Scott and Plata-Salaman, 1999).

An additional complexity to identifying the gustatory connectome is a degree of discrepancy between localized taste-responsive regions in the insular cortex, with responses co-localized with somatosensory stimulation across the anteroposterior extent of the insula and adjacent operculum. Likewise, the piriform cortex shows increased BOLD response to sniffing, the action of inhaling air through the nose, even without the presence of an olfactory stimulus (Kareken et al., 2004; Mainland and Sobel, 2006; Kollndorfer et al., 2014). However, piriform cortex also reacts to trigeminal stimuli, such as carbon dioxide presented to the nasal mucosa, but also to the simple trigeminal component presented in almost all non-pure odorant [e.g., phenyl ethyl alcohol (PEA) or hydrogen sulfide (Bensafi et al., 2007; Albrecht et al., 2010; Pellegrino et al., 2017; Lötsch et al., 2020)]. This can be contrasted to unimodal cerebral cortices responsible for vision, where a large majority of neurons respond to visual stimulation (Hubel and Wiesel, 1965).

This sensory heteromodality should be taken into consideration when conducting seed-based chemosensory connectivity analyses, because a region may not be included in the gustatory connectome simply because it is putative “primary” gustatory cortex; it may just as well be a region included in the “interoceptive” or “oral texture” network. This problem may partially be resolved by supplementing resting-state designs with a sensory task. Employing a task with sensory stimulation allows the construction of subject-specific functional masks that select the most responsive spatial units to stimulation in the relevant sensory modality. Using a subset of sensitive spatial units makes it more likely that fluctuations in neural response are driven by stimulation in the modality of interest. In this case, it may also be preferable to employ DCM models that allow for the specification of driving inputs at stimulus presentation times (Friston et al., 2003) or consider other methods that use betas estimates from task-based designs (e.g., PPI McLaren et al., 2012); BASCO, beta series correlations (Göttlich et al., 2015; Hartig et al., in review). That being said, a connectome derived from primary gustatory areas based on resting-state or structural data may have utility in understanding gustatory neural processing and perhaps also other modalities, like oral somatosensation, as they may have shared underlying mechanisms and are arguably part of the same experiential sensory system of the “oral modality” (Gibson, 1966).

Due to the historical focus of neuroscience on the neocortex (Zilles and Amunts, 2010), less is known about morphological and functional subdivisions in the (para)limbic cortex. This is reflected in some of the more frequently used atlases for automatic anatomical parcellations. For example, the entire insular lobe is one parcellation in the Automated Anatomical Labeling atlas (Tzourio-Mazoyer et al., 2002). Publicly available atlases with parcellations in (para)limbic cortex and subcortical areas include the Brainnetome Atlas (Fan et al., 2016) and the CIT168 Atlas (Pauli et al., 2018).

Coordinates or clusters based on previous studies can be used to functionally define a region-of-interest, but this method will likely bias toward results associated with the specific methods used to collect the data. Meta-analyses like activation likelihood estimation (ALE) maps will lead to more robust functional ROI definitions (Veldhuizen et al., 2011a; Seubert et al., 2013; Yeung et al., 2017; Torske et al., 2021). Particularly promising are ROI’s derived from large dataset connectomic analyses, which has recently been done for the olfactory cortical network with the Human Connectome Project data, as described in more detail above (Arnold et al., 2020).

The choice of atlas is an important one; it will influence the connectome observed, also known as the “atlas concordance problem” (Bohland et al., 2009). Exactly what is the right kind of atlas depends on the research question and the types of inferences researchers want to draw. An illustration of the effects of atlas choice and a framework for choosing between atlases is given by Revell et al. (2021). Selection of appropriate atlases and identification of relevant nodes for chemosensory connectomes requires further methodological study and validation. The optimum choice of atlas for a single study may be somewhat at odds with moving the field forward, since choosing a common atlas by consensus, which enables meta-analyses, can be suboptimal for single studies, when for example the parcellations are not fine enough. A reasonable consensus for the olfactory connectome in humans would be to use the network observed by Arnold et al. (2020) and the publicly available parcellation^1^. If a multimodal analysis of structural and functional data is applied, the inclusion of white matter voxels may severely affect diffusion tensor imaging (DTI) results, which may influence the suitability of atlases and parcellations (Fjaeldstad et al., 2017, 2021). There are currently no such network atlases available for the human gustatory or flavor connectomes (but see Hartig et al. in review for a proposed macaque gustatory connectome).

In summary, the (para)limbic cortices, due to their medial, frontal, and temporal locations suffer from more neuroimaging distortions, due to proximity to sinuses and arteries (as discussed above). This drawback, in combination with relatively sparse neural representation, may lead to a particularly unfavorable (functional) signal-to-noise ratio for chemosensory connectome studies. The use of task over resting-state may be a solution here, as well as optimized data collection techniques that reduce signal distortions and use physiological variables to account for some of the noise. There are only a few appropriate anatomical and functional atlases available for chemosensory regions-of-interest; more research is needed in this area.

### Stimuli

Many chemosensory stimuli may be naively labeled as “gustatory” or “olfactory” when in reality they are multisensory, for example, most odors are also trigeminal and delivery of gustatory stimuli will almost always also include oral somatosensory stimulation. Even worse, some studies label their stimuli as taste or gustatory when in fact the stimuli are flavors (Smits et al., 2007; Vocks et al., 2011), and will additionally stimulate the olfactory system via the retronasal route. Of course, there is a lot to be said for using naturalistic ecological stimuli, but the multisensory nature should be explicitly acknowledged and potential limitations deriving from the multisensory nature would need to be discussed. Connectome studies on the integration of multimodal chemosensory stimuli will need to consider appropriate unisensory baselines. Pure olfactory stimulation can be achieved with careful stimulus selection [e.g., odors confirmed to be pure olfactory by the lateralization method (Croy et al., 2014) or non-discrimination among those who cannot smell (Doty et al., 1978)]. Pure gustatory stimulation may be accomplished with purpose-built gustatory delivery systems that embed a taste bolus into a continuous stream of tasteless solution (Kobayakawa et al., 1999). In other situations, an appropriate choice would be to compare across various stimuli, discounting the common undesired sensory component. The choice for control stimuli in this case is important too, an appropriate baseline would be odorless air or (an individually tailored) tasteless “artificial saliva” solution [i.e., not water, as it may have a taste component (Bartoshuk et al., 1964; Frey and Petrides, 1999; Zocchi et al., 2017)].

Large individual differences in perception mean there is a need to select stimuli or titrate concentration per participant to some psychophysically meaningful level, for example using intensity ratings and matching stimuli to “moderate” intensity. The same procedure may be necessary for control stimuli. For example, an appropriate artificial saliva concentration would be one that “tastes most like nothing” (O’Doherty et al., 2001; Veldhuizen et al., 2007; Baines et al., 2021). The recommendation for matching to a certain target sub- or supra-threshold intensity can be disregarded when the researchers are explicitly interested in the neural correlates of individual differences, in that case the use of an identical stimulus for all participants can actually be desired (see for example Veldhuizen et al. (2020)).

A particular challenge in chemosensory research lies in presenting sufficient repetitions of each stimulus to obtain a reliable estimate of the variable-of-interest. In chemosensory neuroimaging, each stimulus presentation tends to be on the scale of seconds and inter-trial intervals tend to be on the scale of tens of seconds (∼10–15 s for olfactory stimulation and 20–30 s for gustatory stimulation in event-related designs). The need for relatively a lot of time for a single stimulus presentation then restricts the number of different stimuli that can fit into a maximum scanning time of usually 1 h, considered the maximum healthy participants are able to stay focused and comfortable (Szameitat et al., 2009). Thus, the slower rate of stimulus presentation cannot simply be compensated by longer sessions. As a rule-of-thumb, researchers usually aim for ∼20 repetitions per stimulus, which equates to a maximum of 4–5 different stimuli with sufficient repetitions for gustatory studies and maximum of 10 for olfactory studies. The stimulus delivery equipment usually maximizes at a similar capacity. This capacity affects task and sample size (discussed in next section).

### Task

Usually a researcher chooses between resting-state or task design for functional connectivity studies. The absence of a task and the short duration are advantageous for laboratories with minimal equipment who aim to collect data from many participants. Another advantage is the relative absence of movement compared to tasks that require swallowing for example. One disadvantage of the absence of a task is that the results can be difficult to interpret. During resting-state participants’ minds wander freely and they may be experiencing various cognitive and perceptual states. It is unknown how such state differences influence connectivity measures. Recent work suggests that the experience of the participant can be sampled in a standardized manner, which allows time-locked analyses, to deepen insights into networks observed from resting-state data (Gonzalez-Castillo et al., 2021).

Aside from resting-state, researchers may use various kinds of tasks, most commonly a task that addresses a construct of interest, such as a behavior or percept. When comparing task vs. rest in predicting individual differences in fluid intelligence scores, it was shown that a task connectome (based on a variety of tasks) explains over 20% of the variance, while a rest-based connectome explains less than 6% of the variance (Greene et al., 2018). It is unknown if resting-state tasks may explain relatively more variance in chemosensory connectomics, but this seems unlikely given the relatively sparse sensory representation in chemosensory (para)limbic cortices (Smith-Swintosky et al., 1991).

Perhaps related to sparse representation is the possibility that task effects are of a different magnitude relative to sensory stimulation. For instance, searching for a visual stimulus before presentation (Kastner et al., 1999), sniffing odorless air (Zelano et al., 2005) or tasting tasteless solution (Veldhuizen et al., 2007) all increase neural responses in primary sensory cortices. Increased responses in the absence of sensory stimulation reflects an upregulation in baseline activity, thought to be the mechanism by which attention improves detection of weak sensory signals (Luck et al., 1997). Interestingly, Veldhuizen et al. (2007) and Veldhuizen and Small (2011) observed that attentional effects in gustatory and olfactory cortex surpassed actual sensory stimulation in magnitude, which is only around 25% of the sensory signal in the visual modality (Gandhi et al., 1999; Kanwisher and Wojciulik, 2000). This means that neural responses associated with a task employed solely for the purpose of keeping a participant engaged (and from falling asleep) may overshadow any sensory responses in chemosensory studies. The use of labels (“sweet”) or cues (“sniff”) – valid or not – to alert participants to upcoming stimuli will influence neural response and perception (Veldhuizen et al., 2011b). The use of specific tasks to target neural processes of interest (like pleasantness ratings or intensity ratings) will enhance neural responses in different regions (Grabenhorst and Rolls, 2008; Bender et al., 2009). This may be an explicit goal for a connectome study or lead to confounding variables. In general, it is probably unnecessary to prevent participants from falling asleep in studies that use oral stimulation. However, for across-trial variability analyses, it is essential to have a measurement for each trial. In this case, researchers can collect such data during the trial after the sensory neural response is generally modeled (see for example Crouzet et al., 2015).

In chemosensory neuroimaging studies it is important to control for the confounding effects of habituation, the unconscious reduced responsiveness to continuous or repetitive stimulation. The phenomenon is most noticeable for the olfactory system, and it is due to adaptation at both the peripheral (desensitization of olfactory receptors) and central level (decreased activity of the piriform cortex). Reports have shown that habituation depends on the type of odorants, trigeminality, intensity, frequency and timing of the stimulation (Dalton, 2000; Pellegrino et al., 2017). Moreover, odorant molecules do not disappear immediately when exposure ends, but rather are removed from the peri-receptor environment by clearance mechanisms (i.e., nasal submucosal blood flow, nasal mucociliary clearance, and expiratory desorption) (Getchell et al., 1984). Therefore, stimulus presentation must be followed by an adequate odorless airstream. Poellinger et al. (2001) showed different temporal response profiles across brain regions to continuous long (60 s) odor presentation, with the orbitofrontal cortex showing little habituation, the thalamus habituating after 15–30 s, and the piriform cortex habituating within 10–15 s. This means that optimal design choices may be different depending on the region of interest. Similarly, the route of odor presentation is important, as retronasal and orthonasal olfaction are known to be perceptually different (Pellegrino et al., 2021b) and habituate at different rates (Pellegrino et al., 2021b; Xiao et al., 2021). For instance, retronasal odors tend to habituate much slower than orthonasal odor, especially for non-food related odors, and this may be due to activating primary cortices of other modalities bound to the concept of flavor (touch and taste) (Small and Prescott, 2005) as well as the stimuli (a non-food odor in the mouth) feeling foreign (Dalton et al., 2000; Kobayashi et al., 2008). Lastly, chemosensory designs need to intently habituate sensory signals intrinsic to the stimuli, but irrelevant to the task of interest, such as touch. This can, for example, be achieved through a steady stream of the stimulus carrier (i.e., air or artificial saliva), also discussed in the first paragraph of the previous section on “Stimuli”.

To optimize these choices is perhaps more important for chemosensory studies than for other sensory modalities as each stimulus presentation is slow and it is challenging to present many different stimuli (as outlined in the Section “Stimuli” above). For this reason, it is generally recommended to employ block design rather than event-related design. The exception is when researchers are interested in a psychological construct that demands event-related design, for example when studying expectation, or when inter-trial variation is the variable of interest. For olfactory block designs, short runs and a high number of repetitions seems to be advantageous (Han et al., 2020). Block designs are recommended for some connectivity analyses, like DCM (Daunizeau et al., 2011), but there may be no such advantage for other connectivity methods. Advantages of block designs include long enough measurements for many non-time locked connectivity analyses. As block designs tend to be more powerful (Birn et al., 2002), but also potentially lead to more habituation, it may be optimal to alternate between two different stimulus qualities of a similar type [e.g., rose and honeysuckle in floral odor blocks and strawberry and chocolate in food odor blocks; (Sun et al., 2015)]. All of these aspects need to be taken into consideration when setting the proper inter-stimulus or inter-block interval.

Related to the issue of stimulus presentation being relatively slow and the equipment restricting the number of different stimuli (usually 10 max), in chemosensory task design, often a psychological construct of interest is confounded entirely with stimulus identity. An early example is a study that was interested in the neural correlates of food pleasantness that contrasted orally presented chocolate with orally presented salt (Zald et al., 1998). In this case, the stimuli differed in pleasantness, but this was then confounded with differences in olfactory quality (chocolate vs. none) as well as gustatory quality (sweet vs. salty). In a design where one can present many stimuli in a short time-frame, one may include multiple stimuli that are exemplary of a category. For instance, in vision studies, various different images of houses and faces may be used to compare place vs. face processing, then there is much less of a concern for confounding by, for example, a tree branch being in front of the house in one of the images or the configuration of windows and door resembling a face in another one of the images. Therefore, task design in chemosensory neuroimaging needs to consider careful matching of stimuli across tasks if stimulus identity is the manipulated variable.

Summarizing, when designing chemosensory connectome studies additional attention should be given to: (1) whether to use a task or not; (2) timing of the task relative to the time-window-of-interest for measuring neural response; (3) habituation; and (4) confounding task with a stimulus.

### Acoustic Noise and Visual Input

Depending on the type of neuroimaging modality, potential sensory confounders may be systematically introduced. In fMRI studies, chemosensory data acquisition is inevitably accompanied by loud noises produced by the scanner, i.e., 99–125 dB (Hattori et al., 2007), with peak noise up to 138 dB (Ravicz et al., 2000). While some types of background noise have been shown to disrupt olfactory performance (Seo et al., 2011), fMRI-specific acoustic noise has only been found to reduce the olfactory threshold score without affecting discrimination, identification, identification certainty, hedonic rating, or intensity rating (Fjaeldstad et al., 2019). Similarly, acoustic fMRI noise had no effect on taste perception, including identification, the certainty of identification, perceived intensity, and hedonic rating (Lorentzen et al., 2021), but also see (Yan and Dando (2015), Havermans and Hendriks (2020)). A recent study shows how resting state network connectivity is reduced by fMRI acoustic noise, as compared to connectivity estimated with MEG (Pellegrino et al., 2022). The potential effects of acoustic fMRI-noise on the functional connectome in chemosensory studies have not yet been investigated. Nevertheless, efforts have been made to acclimatize subjects to the acoustic noise associated with magnetic resonance scanners, with mock scanning sessions common in human imaging, particularly in children (O’Rawe et al., 2019), as well as habituation training for scanning in awake rodents (Winkelmeier et al., 2021).

As many chemosensory studies do not present visual stimuli, participants may not be required to keep their eyes open. Participants may also be instructed explicitly on whether to keep their eyes open or closed. A recent study showed that when placing a seed in primary visual cortex, different connectivity patterns are observed with this region under eyes-closed vs. eyes-open instructions (Costumero et al., 2020). Specifically, greater connectivity among default mode and sensorimotor networks was observed when subjects’ eyes are closed, with higher salience network connectivity observed when subjects’ eyes are open. This suggests that opening or closing the eyes induces an interoceptive or exteroceptive state for functional connectivity. Indeed, this may be particularly relevant for chemosensory perception as chemo-senses are arguably an intermediate between the interoceptive and exteroceptive senses. In agreement with this, Wiesmann et al. (2006) showed that olfactory and gustatory areas specifically show increased activation under eyes-closed conditions. Chemosensory studies may prefer to use eyes-closed conditions. We recommend providing clear instructions toward eye closure for participants and verifying compliance and incorporating regressors (Brodoehl et al., 2016).

These studies on auditory noise and visual input show how the choices for baseline state (regardless of control or baseline stimuli) may have a considerable impact on activation and connectivity patterns.

### Data Cleaning/Model Fitting

Many data cleaning and model fitting conventions are established in studies using the visual modality. However, we know that sensory and perceptual processing in the chemical senses generally follows a different timescale than other sensory modalities [as reflected in reaction times (Cain, 1976; Posner et al., 1980; Halpern, 1986)]. The slower processing is presumably driven by central nervous system (CNS) events as the receptor mechanisms are thought to operate on a different timescale (Torre et al., 1995). If CNS processing of chemosensory stimuli is slower than vision, it is unclear how appropriate various conventional modeling choices are for chemosensory studies. Despite these known differences between sensory modalities, data cleaning and modeling choices in chemosensory neuroimaging usually follow the conventions that were developed for vision research. Examples of such models include the canonical hemodynamic response function (HRF) for functional localization studies, and deconvolution of the HRF for PPI analysis (Gitelman et al., 2003) and the various models incorporated into DCM (Friston et al., 2003). Other connectivity analysis methods usually use a more direct approach of correlating time series that are not influenced by such choices, as no model is applied. Note that the use of models is independent of the use of task, one may still use a task or sensory stimulation to perturb brain activity to improve sensitivity to detecting a network without explicitly modeling those perturbations.

However, there are a few adaptations that can easily be taken into account by chemosensory researchers. For example, the use of high-pass filters during data cleaning should be adjusted to the slower stimulus presentation or task blocks customary in chemosensory studies. The recommended cut-off time is 1.5× the period between subsequent presentations of the same event-of-interest (Jezzard et al., 2001). In a block design with 20 s rest between blocks and 60 s blocks and 4 different blocks in a run, this may lead to a 300 s high-pass filter, a significant departure from the convention of 128 s in for example the SPM toolbox. For fitting GLM models it is appropriate to include temporal derivatives to deal with potentially slower hemodynamic responses, which is a reasonable expectation in chemosensation. Methodological studies are needed to optimize these data analysis choices for chemosensory neuroimaging in general and chemosensory connectome studies specifically. However, for information about temporal aspects of processing, researchers may also look to fundamental EEG and MEG studies with translational applicability (Crouzet et al., 2015; Iravani et al., 2020).

### Sample Size Issue and Brain-Behavior Relation

Sample size is a critical issue for all chemosensory studies, since fewer participants can usually be recruited relative to other sensory modalities (given similar constraints, limiting financial, manpower, and other resources for studies), as a trial in an event-related fMRI study in which a visual stimulus is presented will take around 4–6 s, and between 10–30 s for chemosensory stimuli. This means that in chemosensory studies the number of stimulus presentations is usually relatively low, which presumably affects within-participant reliability negatively. Likewise, this same constraint negatively influences the number of participants in a study. Fortunately, the median sample size of taste and food fMRI studies significantly increased from 11.5 to 35.5 from the early 2000s to the end of the 2010s (Yeung et al., 2020). At one time it was estimated that 16 subjects would be adequate for detecting a medium effect size from task-based fMRI experiments (Friston, 2012). However, a sample size of even 30 can be deemed too small for replicable results in the current perspective, as empirical studies have since recommended at least 50 or even 100 for task-based fMRI results (Turner et al., 2018; Bossier et al., 2020) and 70+ participants for functional connectivity results (Sideridis et al., 2014). For stable estimates of correlations between BOLD response and cognitive task performance, roughly 80 or more participants are needed (Grady et al., 2021). Instead of relying on these rules-of-thumb, special consideration should be given when within-participant variance is relatively big. Getting sufficient individual-level data, such as by longer durations of resting-state fMRI or more repetitions of events for task-based fMRI experiments, may stabilize the parameter estimates and hence improve replicability (Nee, 2019). But as noted above, minimizing within-participant variance in chemosensory studies is difficult given the constraints placed on the number of stimulus repetitions by long trials. Meanwhile, between-participant confounding factors should also be addressed carefully as they may modulate cerebral processing of chemosensory perception, such as age (Hoogeveen et al., 2015), gender (Yeung, 2018), handedness (Cerf et al., 1998; Faurion et al., 1999), body mass index, and hunger (Chen and Zeffiro, 2020). In case of social chemosensory signals (e.g., human axillary sweat), other confounding factors should be considered, including menstrual cycle phase and oral contraceptive use, as they can also influence chemosensory perception (Derntl et al., 2013; Endevelt–Shapira et al., 2020), but see also Schaefer et al. (2021) for negative results. Given the influences of sex-specific and social cues on chemosensory perception, special detail to the inclusion of balanced sexes in a study cohort should be considered. Studies including all male or female participants should be cautious about generalizing findings across genders until a complementary study is conducted with consideration of potential gender or sex-specific differences. Solutions to the sample size conundrum for the chemosensory studies may lie in data-pooling (facilitated by consensus in reporting and methodology as well as data sharing) and Many Labs style studies (in which multiple labs collect data for the same study), options that will also be discussed in the next section.

## Best Practices From the Connectomics Field

Researchers studying the connectomics of chemosensory perception should look at both the disciplines of connectomics and chemosensory neuroimaging for best practices. Brain connectomics is a relatively large and fast advancing field, with rapidly changing conventions. Here, we summarize the relevant best practices from neuroimaging in general and connectomics specifically.

### Pre-analysis Declaration of Hypotheses and Analysis Plan

Since connectomics studies include many regions and connections, there is a greater likelihood of observing false-positives. Hypothesizing (only) after the results are known, or changing the hypothesis *post hoc* (i.e., “HARKing”) is a well-known bad practice used to avoid correcting for multiple comparisons and biases toward false positives.

One best practice is to -- before data collection -- create a public, time-stamped, non-editable document of the study’s hypotheses, sample size and analysis plan, known as a pre-registration. The study team creates the pre-registration and there is no peer-review process or journal involved. Pre-registration can also be done after data collection (but before data analysis) or for exploratory analyses without a hypothesis. There are templates for fMRI study pre-registration, for instance, available to the community via the Open Science Framework^2^. The goal is to provide a transparent, time-stamped, unalterable account of the original plan before data analysis to justify regions-of-interest and reduced number of comparisons. This account can then be cited in a manuscript submitted for publication and easily inspected by reviewers, editors, and readers. The field of human chemosensory perception and neuroimaging has recently shown an accelerated pace in the adoption of these practices (Havermans and Hendriks, 2020; Parma et al., 2020; Iravani et al., 2021b; Thunell et al., 2021; Torske et al., 2021). In absence of a pre-registration, an alternative solution is to be transparent about *post hoc* analyses and to perform the appropriate corrections for multiple comparisons.

Another option is to create a registered report. While this sounds similar to pre-registration and has some commonalities, there are some critical differences. Registered reports are peer-reviewed data collection plans that can be provisionally accepted for publication by a journal (currently by a limited number of journals). This must be done before data collection starts. An accepted registered report will be published by the same journal regardless of the observed results. In the fields of psychology and neuroscience, registered reports were shown to outperform non-registered peer-review publications in methodological rigor, analysis, and overall paper quality (Soderberg et al., 2021).

Note that pre-registrations and registered reports in and of themselves cannot prevent scientific malpractice. Ultimately, part of the solution against HARKing has to come from eliminating publication bias against null effects. We encourage journals and their editors to explicitly commit to publishing null results that are supported by appropriate equivalence statistics, as was done for example in the paper by Pellegrino et al. (2021a) demonstrating a lack of difference in neural response to odors in piriform cortex in post-traumatic brain injury.

### Reporting and Data Analysis Guidelines

The Committee on Best Practices in Data Analysis and Sharing (COBIDAS) of the Organization for Human Brain Mapping (OHBM) has formulated a set of reporting guidelines for data analyses and data sharing of human neuroimaging studies https://www.humanbrainmapping.org/files/2016/COBIDASreport.pdf. This report introduced a standard terminology (e.g., what should be understood as a ‘run’ and what as a ‘session’), minimum reporting standards with a particular focus on analysis and data sharing. It is also accompanied by a very practical checklist for researchers to complete while writing the methodology section of a manuscript (Nichols et al., 2017). They also include a paragraph specifically on connectivity analyses:

“The critical issues when reporting functional connectivity differ between types of approaches, for example exploratory multivariate vs. seed-based correlation methods, which provide whole maps, versus confirmatory multivariate methods for a handful of regions. When reporting multivariate decomposition methods like ICA or PCA, state how the number of components were selected. With either ICA or seed-based analyses, when conducting inference on multiple networks, be sure to account for multiplicity when searching over the networks. For example, if testing for patient vs. control differences in the default mode, attentional, visual, and motor networks, the inference must account for not only the voxels within networks, but additionally for searching the four IC maps for significance.”

Of particular interest are the following COBIDAS expansions that are currently in development. The first is the Best Practices on Large-Scale Brain Network Nomenclature. Connectomics currently lacks a consistent network taxonomy. For example, a network containing the insula may be referred to as “cingulo-opercular network,” “salience network,” or “ventral attention network.” This inconsistency hinders future cross-study comparisons and meta-analyses. A labeling scheme based on anatomical labels has been proposed (Uddin et al., 2019), but has not yet been finalized.

Second, e-COBIDAS is in development. In this app, researchers choose descriptors from drop-down lists to describe a study’s experimental design, and then a “boilerplate” methods and analysis section is automatically generated. In e-COBIDAS, there are detailed choices for stimuli and task design that COBIDAS does not address. These expansions that are in development are usually crowd-sourced initiatives with the explicit solicitation of input of expertise from a wide variety of researchers. We encourage researchers in the chemosensory modalities to contribute to the development of these guidelines, as researchers working in the chemical senses are generally underrepresented in the community responsible for guideline development. For example, visual and auditory research with computer-generated stimuli uses entirely different reporting parameters from chemosensory stimuli that are prepared wet-lab style and where presentation is controlled with different parameters. At minimum (and this is not an exhaustive list), concentration, flow rate, volume, temperature, humidity, intertrial intervals, baseline/control stimuli, stimulus matching and/or titration should be reported. Tools meant to standardize reporting of stimulus presentation methodology that overlook all these relevant parameters cannot be adopted by the chemosensory field, which hinders the field’s ability to keep up with general advances in the neuroimaging community. We recommend that the chemosensory neuroimaging community gets involved with the development of such tools. Some of the best practices relevant to the neuroimaging of food also apply to chemosensory studies; this recent publication by Smeets et al. (2019) includes a useful list of confounding factors.

### Big Data, Open Data, and Meta-Analyses

Large, publicly available datasets, like the Human Connectome Project (HCP) and the UK Biobank, have emerged as important resources in the field of human connectomics. A recent review gives an excellent perspective on the challenges and progress in conducting reproducible large-scale connectivity data analyses, including important recommendations regarding data management and ethical considerations (Laird, 2021). We would like to note that these large publicly available datasets rarely include chemosensory measures, with the exception of a subset of the HCP data, which include some of the olfactory and gustatory measures from the NIH toolkit (Coldwell et al., 2013; Dalton et al., 2013). The field would benefit greatly from the inclusion of (more) chemosensory measures in big neuroimaging data projects. This will likely require the chemosensory neuroimaging community gaining a stronger foothold in the general neuroimaging community, which includes the recognition of the importance of the chemical senses. Alternatively, the chemosensory neuroimaging community can start their own big data initiative by running distributed studies in a “Many Labs” model of collaboration (Stroebe, 2019).

In addition, the chemosensory neuroimaging community can likely book more progress if consensus is developed on reporting and data analysis guidelines and more data is shared. Sharing of raw neuroimaging data can be complex in relation to data privacy concerns, but if allowed, there are good depositories available (see Table 2). If local legislation prevents the sharing of raw data, group-level analysis maps can also be shared. All these initiatives facilitate the conduct of meta-analyses, the best scientific tool to systematically assess evidence across studies.

**TABLE 2:** Best practices from the field of neuroimaging studies as they apply to chemosensory connectome neuroimaging studies.

Best practice label	Details	Source
Reporting guidelines for stimulus and task design (general)	• Drop-down lists of choice for mandatory reporting items. • Generates boilerplate methods sections.	e-COBIDAS
Reporting guidelines for chemosensory stimulus selection and task design	• Details of multisensory nature of stimuli and address confounding factors. • Optimize task.	Current paper, Best practices food-related neuroimaging
Reporting guidelines for confound adjustment and filtering in data analysis	• Method for detecting movement artifacts, movement-related variation, and remediation. • Use of global signal regression, exact type of global signal used and how it was computed. • Whether a high- or low- pass temporal filtering is applied to data, and at which point in the analysis pipeline.	COBIDAS
Reporting guidelines for connectivity analyses	• Exploratory multivariate vs. seed-based correlation methods. • ICA or PCA: how many components? • Multiple comparison correction for multiple networks.	COBIDAS
Data labeling and organization	• Standardized file naming and folder organization. • Sidecar files with data collection parameters.	BIDS
Data sharing	• Citable. • Organized. • Permanent. • Can be used in meta-analyses.	Openneuro, NeuroVault.

## Conclusion and Future Directions

Network-like approaches describing neuronal processes via functional mechanisms and interactions between brain regions are getting more and more attention within neuroscience. With this guide, we hope to raise awareness of the importance of these approaches in the field of chemosensory perception, given the specific physiology of chemosensory modalities and the multisensory nature of ecological chemosensory stimuli. Indeed, there are established techniques that have not yet been rigorously applied to imaging studies in the chemical senses, leaving insights to sensory processing overlooked. Therefore, this article aimed to communicate current existing neuroimaging and connectome guidelines to the chemosensory community and to outline specific challenges for chemosensory studies, in effect: (a) physiological noise, (b) movement, (c) the correct use of stimuli, (d) task design, (e) acoustic noise, (f) data cleaning and modeling fitting, and sample size issues. Finally, we offered some suggestions to improve the quality of the research data and make results more meaningful and replicable: (1) optimized study designs, (2) reporting guidelines, (3) consensus on brain parcellations, (4) consortium research, and (5) data sharing.

## Data Availability Statement

The original contributions presented in the study are included in the article/supplementary material, further inquiries can be directed to the corresponding author.

## Author Contributions

MV, CC, and FF wrote the first draft of the manuscript. All authors wrote sections of the manuscript, contributed to manuscript revision, read, and approved the submitted version.

## Conflict of Interest

The authors declare that the research was conducted in the absence of any commercial or financial relationships that could be construed as a potential conflict of interest.

## Publisher’s Note

All claims expressed in this article are solely those of the authors and do not necessarily represent those of their affiliated organizations, or those of the publisher, the editors and the reviewers. Any product that may be evaluated in this article, or claim that may be made by its manufacturer, is not guaranteed or endorsed by the publisher.

## Footnotes

1
https://github.com/LiLabFSU/olfactoryRSN

2
https://osf.io
